# Severe Aortic and Mitral Regurgitation Secondary to Infective Endocarditis Presenting as Acute Respiratory Failure

**DOI:** 10.7759/cureus.107578

**Published:** 2026-04-23

**Authors:** Mehul Mange, Deepak Sharma, Mahesh Eddula, Priyanka Sharma

**Affiliations:** 1 Anesthesia and Critical Care, Macclesfield District General Hospital, Macclesfield, GBR; 2 Critical Care Medicine, Sandwell and West Birmingham NHS Trust, Birmingham, GBR; 3 Research and Development, University of Birmingham, Birmingham, GBR

**Keywords:** acute respiratory failure (arf), infectious disease medicine, infective endocarditis, invasive mechanical ventilation, valvular-heart disease

## Abstract

Infective endocarditis (IE) typically presents with fever, a new or changing cardiac murmur, and embolic phenomena. Acute respiratory failure (ARF) as the dominant presenting feature, without overt cardiac signs, is uncommon and creates significant diagnostic challenges. We present a previously healthy 24-year-old man who attended the Emergency Department with fever, productive cough, and ARF requiring mechanical ventilation for bilateral pulmonary infiltrates. Cardiovascular examination on admission was unremarkable: there was no audible cardiac murmur and no peripheral stigmata of IE. Blood cultures subsequently yielded *Staphylococcus aureus*. Following initial extubation on day 4, the patient deteriorated rapidly within 12 hours, necessitating reintubation. The emergence of a new diastolic murmur prompted urgent echocardiography, revealing large mobile vegetations on both the aortic and mitral valves with severe regurgitation of each, consistent with left-sided IE. Definite IE was confirmed by two major modified Duke criteria. Emergency double mechanical valve replacement was performed on day 6, with the patient discharged on day 14 on lifelong anticoagulation. This case illustrates that acute severe valvular regurgitation in IE can precipitate cardiogenic pulmonary edema closely mimicking acute respiratory distress syndrome (ARDS). Concomitant aortic and mitral valve involvement likely resulted from contiguous spread via the aorto-mitral curtain. Crucially, the absence of a murmur or peripheral stigmata of IE on initial examination did not exclude the diagnosis. Early echocardiographic evaluation in unexplained ARF is paramount, particularly in the context of staphylococcal bacteremia.

## Introduction

Infective endocarditis (IE) is a life-threatening infection of the cardiac endothelium, most frequently involving the native heart valves, associated with an in-hospital mortality of 15-30% despite advances in antimicrobial therapy and surgical management [1]. The classical presentation comprises persistent fever, a new or changing cardiac murmur, and embolic or immunological phenomena. Atypical presentations, however, are well recognized and may cause critical diagnostic delays [2,3]. Respiratory compromise in left-sided IE arises most commonly from cardiogenic pulmonary edema secondary to acute severe valvular regurgitation; in right-sided disease, septic pulmonary emboli predominate [4]. Pulmonary symptoms are a recognized consequence of hemodynamically significant valvular regurgitation. However, IE presenting primarily as acute respiratory failure (ARF) mimicking acute respiratory distress syndrome (ARDS), without any cardiac signs, is rare and has been described largely in isolated case reports and small case series, making it diagnostically hazardous [5,6].

A critical pitfall in such presentations is the application of standard sepsis resuscitation protocols with aggressive intravenous fluid administration, which, specifically in the context of unrecognized acute severe valvular dysfunction, can precipitate or exacerbate cardiogenic pulmonary edema [3]. The diagnostic challenge is compounded when concomitant conditions, such as active Epstein-Barr virus (EBV) infection, offer an alternative explanation for the clinical picture, further delaying cardiac evaluation [7,8].

We describe a case of concurrent severe aortic and mitral regurgitation secondary to left-sided IE presenting primarily as ARF without overt cardiac signs on admission. The case underscores the indispensable role of early echocardiographic evaluation in unexplained respiratory failure and advocates for routine cardiac assessment within sepsis management algorithms, particularly in the context of staphylococcal bacteremia.

## Case presentation

A 24-year-old man with no significant past medical history, a body mass index of 23 kg/m², no cardiac risk factors, no history of rheumatic fever, and no relevant family history presented to the Emergency Department with a three-day history of acute-onset dyspnea, productive cough, and fever. He was a moderate cigarette smoker, denied illicit substance use or intravenous drug use, was not taking any regular medications, and had no known drug allergies.

In the weeks preceding his admission, the patient had experienced unintentional weight loss, drenching night sweats, and generalized malaise. As part of a lymphoma workup for left cervical lymphadenopathy, he had undergone a left cervical lymph node biopsy and positron emission tomography-computed tomography (PET-CT) scan approximately two weeks before presentation. Histology and imaging were consistent with reactive viral lymphadenitis. Serology confirmed EBV IgG positivity with a markedly elevated EBV polymerase chain reaction (PCR) viral load, indicative of active infection at the time of admission. No outpatient cardiac assessment had been performed during this period of constitutional illness.

On general examination, the patient was acutely distressed, tachypneic at 30-34 breaths per minute, and speaking in short phrases. Oxygen saturation was 84% on 60% oxygen delivered via a Venturi mask. Vital signs revealed a temperature of 38.6°C, heart rate of 108 beats per minute, and blood pressure of 138/68 mmHg. Lung auscultation revealed globally reduced air entry with widespread bilateral crepitations. Cardiovascular examination was unremarkable: there was no audible cardiac murmur on auscultation, no raised jugular venous pressure, no peripheral edema, and no peripheral stigmata of IE, including Janeway lesions, Osler nodes, splinter hemorrhages, or Roth spots. Abdominal examination was unremarkable with no organomegaly.

Initial arterial blood gas analysis on 60% inspired oxygen confirmed Type 1 (hypoxemic) respiratory failure, with a PaO₂/FiO₂ ratio of 90 mmHg, consistent with severe hypoxemia; after intubation, the PaO₂/FiO₂ ratio met Berlin criteria for severe ARDS. N-terminal pro-B-type natriuretic peptide (NT-proBNP) was not measured on admission, which, in retrospect, would have assisted in differentiating an ARDS-predominant from a cardiogenic-predominant picture. Laboratory investigations on admission are summarized in Table 1. These demonstrated a markedly elevated inflammatory response, including leucocytosis, neutrophilia, C-reactive protein (CRP) of 180 mg/L, elevated erythrocyte sedimentation rate (ESR), and procalcitonin of 4.2 ng/mL, consistent with bacterial sepsis. Troponin was within normal limits. An extended respiratory panel, including Legionella and pneumococcal urinary antigens, *Pneumocystis jirovecii *PCR, and hepatitis B and C serology, was all negative. 

**Table 1:** Laboratory investigations on admission CRP: C-reactive protein; ESR: erythrocyte sedimentation rate; CK-MB: creatine kinase-MB; eGFR: estimated glomerular filtration rate; PCR: polymerase chain reaction; NT-proBNP: N-terminal pro-B-type natriuretic peptide

Parameter	Result	Unit	Reference Range
Hemoglobin	10.0	g/dL	13.5-17.5 g/dL
White cell count	18.6	×10⁹/L	4.0-11.0 ×10⁹/L
Neutrophils	88	%	40-75%
Lymphocytes	7	%	20-45%
Monocytes	3	%	2-8%
Eosinophils	2	%	1-6%
C-reactive protein (CRP)	180	mg/L	<5 mg/L
Erythrocyte sedimentation rate (ESR)	65	mm/hr	<20 mm/hr
Procalcitonin	4.2	ng/mL	<0.5 ng/mL
Creatinine	85	µmol/L	60-110 µmol/L
eGFR	>90	mL/min/1.73m²	>90 mL/min/1.73m²
Alanine aminotransferase (ALT)	35	U/L	7-56 U/L
Aspartate aminotransferase (AST)	40	U/L	10-40 U/L
Bilirubin (total)	15	µmol/L	5-17 µmol/L
Troponin I	<0.04	ng/mL	<0.04 ng/mL
CK-MB	2.1	ng/mL	0.5-3.6 ng/mL
Legionella urinary antigen	Negative	—	Negative
Pneumococcal urinary antigen	Negative	—	Negative
Hepatitis B & C serology	Negative	—	Negative
*Pneumocystis jirovecii *PCR	Negative	—	Negative
HIV 1/2 serology	Negative	—	Negative
NT-proBNP	Not measured on admission	—	(<125 pg/mL if <75 yrs)

**Figure 1:**
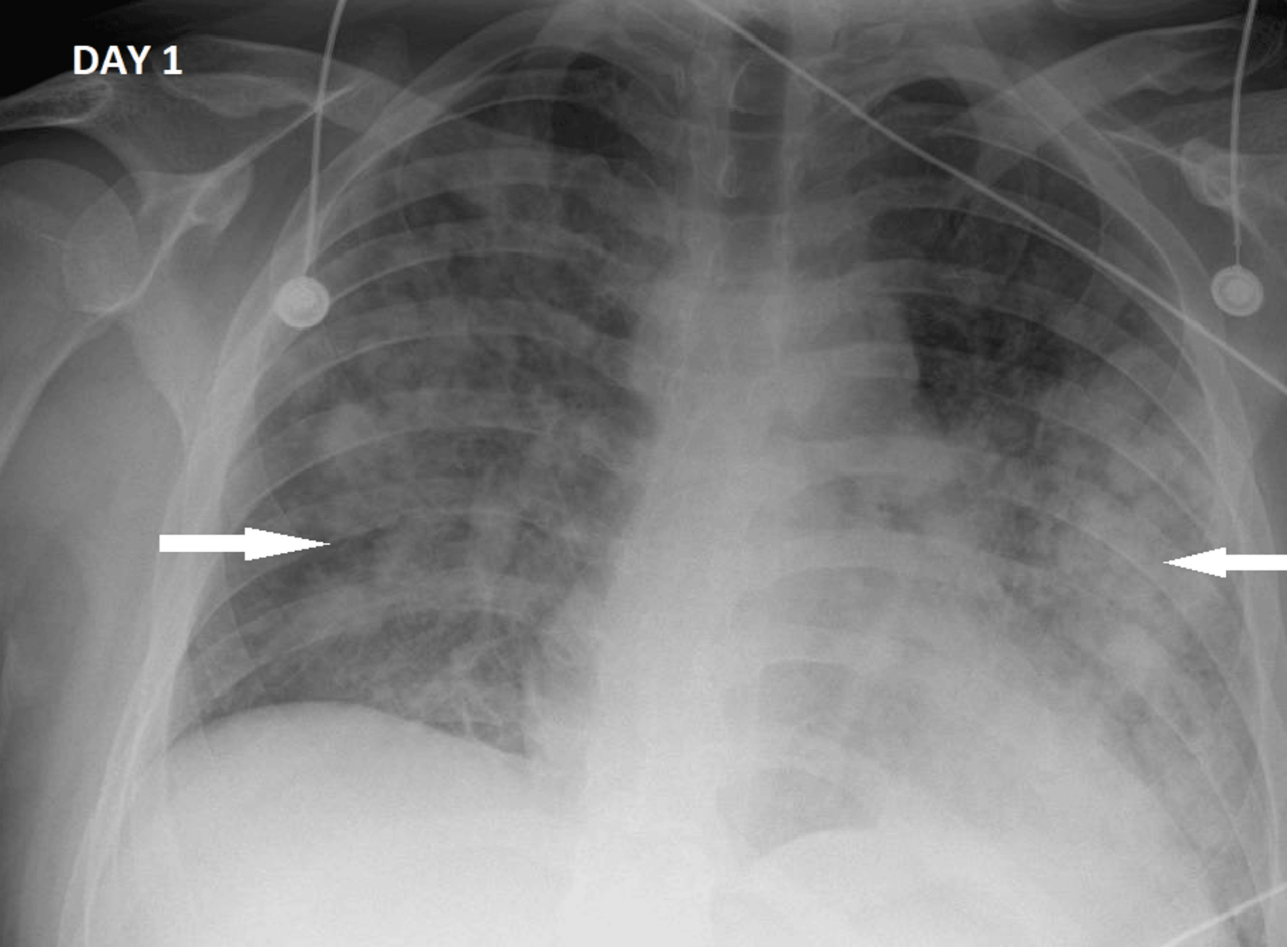
Anteroposterior chest radiograph obtained on day 1 (day of admission) Demonstrating bilateral, diffuse pulmonary infiltrates (white arrows) consistent with acute respiratory failure.

Chest radiography demonstrated bilateral pulmonary infiltrates (Figure 1), in keeping with ARDS. The patient was managed for presumed ARDS secondary to viral infection with concurrent bacterial pneumonia. Despite empirical intravenous piperacillin-tazobactam and supportive care, his respiratory status deteriorated, and he required endotracheal intubation. Invasive mechanical ventilation was conducted using a lung-protective strategy. Tidal volumes were ≤6 mL/kg predicted body weight, and plateau pressures were maintained below 30 cmH₂O. High levels of positive end-expiratory pressure (10-14 cmH₂O) were required due to a persistently low PaO₂/FiO₂ ratio. Hemodynamic instability developed, necessitating low-dose norepinephrine support (less than 0.25 µg/kg/min) to maintain mean arterial pressure. Blood, urine, and sputum cultures were obtained on admission.

Blood cultures drawn on admission yielded *Staphylococcus aureus* on day 2; antibiotic therapy was accordingly rationalized to intravenous flucloxacillin 2 g every six hours following microbiological advice, with sensitivities confirming meticillin-sensitive *S. aureus* (MSSA). Urine and sputum cultures returned negative. The patient demonstrated clinical and radiological improvement and was successfully extubated on day 4.

**Figure 2:**
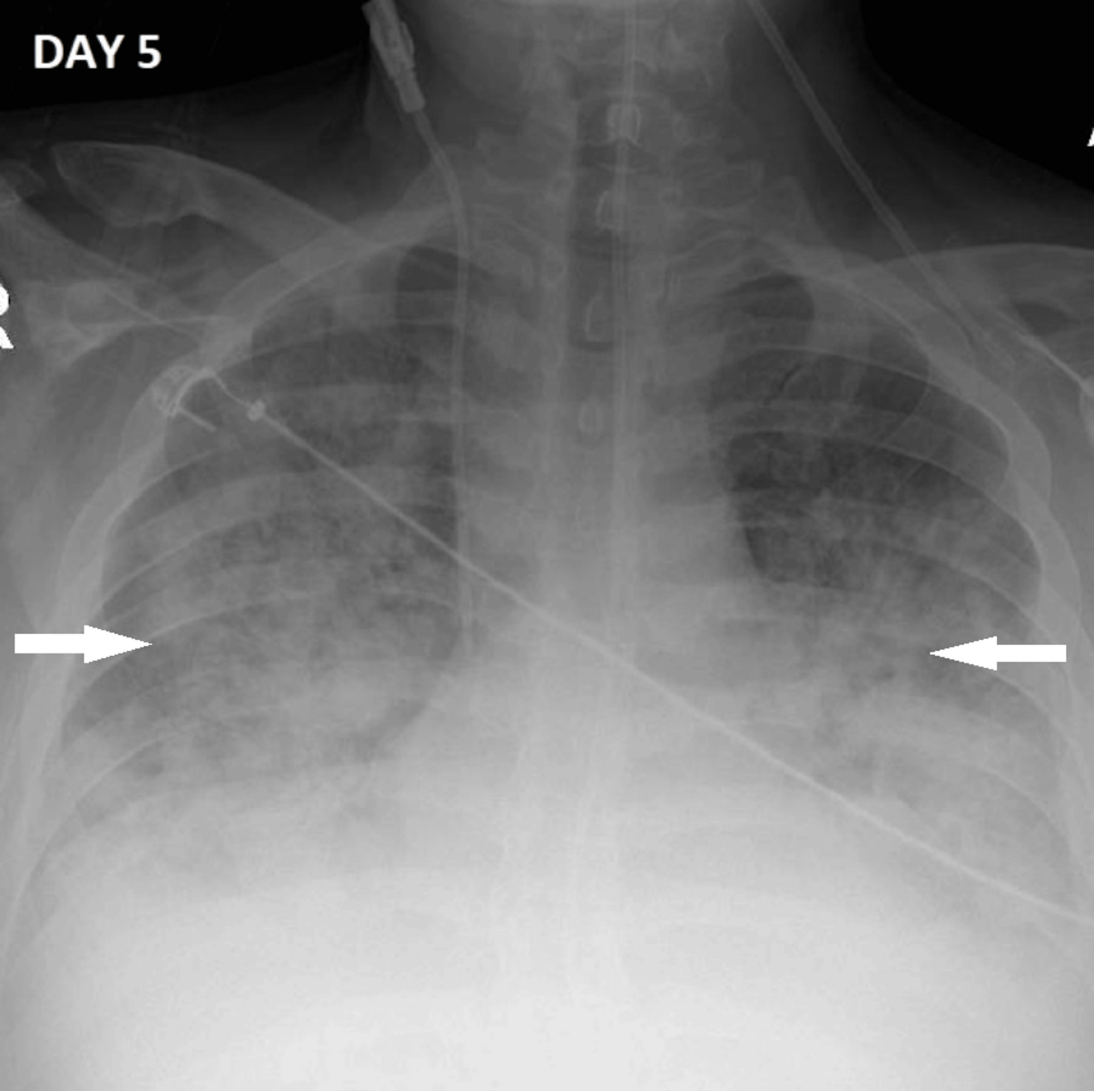
Anteroposterior chest radiograph obtained on day 5, prior to reintubation Demonstrating marked worsening of bilateral pulmonary infiltrates and airspace opacification (white arrows) consistent with cardiogenic pulmonary edema. Nasogastric tube, central venous catheter, and peripheral lines are in situ.

Within 12 hours of extubation, the patient developed acute respiratory deterioration with rapidly escalating oxygen requirements. He required sequential escalation through nasal high-flow oxygen and continuous positive airway pressure (CPAP) before reintubation became necessary. At reintubation, the PaO₂/FiO₂ ratio had fallen to 86 mmHg. A repeat chest radiograph at this juncture demonstrated a marked deterioration with worsening bilateral pulmonary edema (Figure 2). Critically, repeat cardiovascular auscultation now revealed a new early diastolic murmur at the left sternal edge and a pansystolic murmur at the cardiac apex, findings absent on initial examination. These new auscultatory findings, in the context of confirmed staphylococcal bacteremia, immediately prompted urgent echocardiographic assessment.

**Figure 3:**
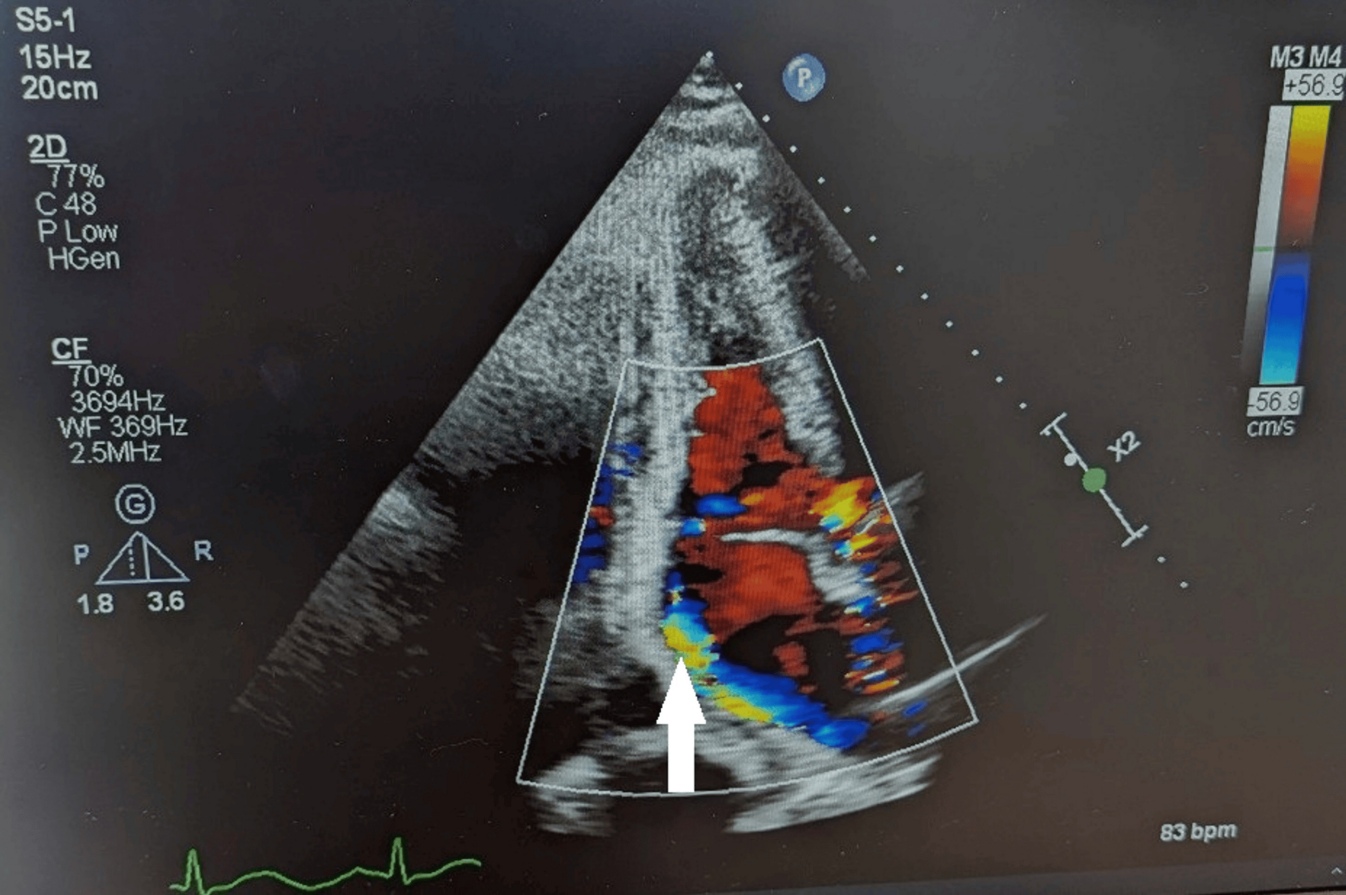
TTE, apical two-chamber view, with colour flow Doppler (Nyquist limit 56.9 cm/s) Demonstrating a large eccentric mitral regurgitation (MR) jet (mosaic colour signal- marked as white arrow) filling the majority of the left atrium (LA) during systole, consistent with severe MR. LV: left ventricle; LA: left atrium; MV: mitral valve. Large mobile vegetations on the aortic and mitral valves were identified on other acquired views and confirmed on transoesophageal echocardiography (TTE).

Urgent bedside transthoracic echocardiography (TTE) was performed. In the parasternal long-axis view, color flow Doppler (Nyquist limit 40 cm/s) demonstrated a broad eccentric jet of severe aortic regurgitation (AR) extending from the aortic valve into the left ventricular outflow tract. In the apical two-chamber view, color flow Doppler revealed a large eccentric jet of severe mitral regurgitation (MR) directed into the left atrium (Figure 3). Large, mobile vegetations were identified on both the aortic and mitral valves. Quantitative echocardiographic assessment confirmed severe AR (effective regurgitant orifice area (EROA) of 0.35 cm²; regurgitant fraction of 55%) and severe MR (EROA of 0.45 cm²; regurgitant fraction of 60%) [9]. Left ventricular ejection fraction was preserved at 55%. Left atrial enlargement was present (volume index 45 mL/m²), with elevated pulmonary artery systolic pressure at 55 mmHg and preserved right ventricular function. Trans-oesophageal echocardiography (TOE) confirmed these findings and excluded perivalvular abscess formation or fistula.

The diagnosis of definite IE was established on the basis of two major modified Duke criteria: positive blood cultures with *Staphylococcus aureus*, a typical microorganism for IE, from two separate blood culture sets; and echocardiographic evidence of endocardial involvement, including large mobile vegetations on both aortic and mitral valves with severe regurgitation [10]. These two major criteria alone confirm the diagnosis of definite IE without requiring additional minor criteria.

Following immediate multidisciplinary team discussion involving critical care, cardiology, microbiology, and cardiothoracic surgery, the patient fulfilled a Class I indication for urgent surgery per the 2023 European Society of Cardiology (ESC) Guidelines for the management of IE: refractory heart failure caused by severe valvular regurgitation [2]. He was urgently transferred to a tertiary cardiothoracic center on day 6. Emergency double valve replacement was performed successfully. Intraoperatively, friable vegetations and extensive leaflet destruction of both the aortic and mitral valves were confirmed, consistent with active IE. In view of the patient's young age and anticipated longevity, bilateral mechanical valve prostheses were implanted. This necessitates lifelong anticoagulation with warfarin. Histopathological examination of the excised valvular tissue confirmed acute inflammation with bacterial colonization.

The patient had an uneventful postoperative recovery with progressive weaning from ventilatory support and complete resolution of respiratory symptoms. He was discharged on day 14 on a six-week course of intravenous flucloxacillin and warfarin with a target international normalized ratio (INR) of 2.5-3.5 and was scheduled for regular cardiology and anticoagulation clinic follow-up. A summary timeline of key clinical and therapeutic milestones is presented in Table 2.

**Table 2:** Timeline of key clinical events TTE: transthoracic echocardiography; TOE: trans-oesophageal echocardiography; AR: aortic regurgitation; MR: mitral regurgitation; IE: infective endocarditis; IV: intravenous; INR: international normalised ratio; EBV: Epstein-Barr virus; PET-CT: positron emission tomography-computed tomography

Day / Time	Event
Two weeks prior	Left cervical lymph node biopsy and PET-CT scan; histology consistent with viral lymphadenitis
Ten days prior	EBV IgG confirmed positive, with a high EBV PCR viral load indicating active infection
Day 0	Presentation with acute dyspnea, productive cough, and fever. Admission bloods, cultures, and chest radiograph were performed. Cardiovascular examination unremarkable. Intubation and mechanical ventilation were initiated for Type 1 respiratory failure
Day 1	The extended respiratory panel returned negative. Empiric intravenous piperacillin–tazobactam commenced
Day 2	Blood cultures were positive for *Staphylococcus aureus*. Antibiotic therapy de-escalated to IV flucloxacillin 2 g six-hourly
Day 3	Hemodynamic support with low-dose norepinephrine was initiated
Day 4	Extubation following clinical improvement
Day 5	Rapid respiratory deterioration within 12 hours of extubation; a new diastolic murmur was identified on auscultation. Reintubation. Urgent bedside TTE: severe AR and MR with large vegetations on the aortic and mitral valves. TOE confirmed findings and excluded an abscess. Definite IE confirmed per modified Duke criteria (2 major)
Day 6	Emergency transfer to the tertiary cardiothoracic centre. Successful double mechanical valve replacement
Day 14	Discharged on a six-week course of IV flucloxacillin and lifelong anticoagulation (warfarin, target INR 2.5–3.5). Cardiology and anticoagulation clinic follow-up arranged

## Discussion

The initial absence of a cardiac murmur and peripheral stigmata of IE on admission is a well-recognized but under-appreciated phenomenon in acute-onset left-sided IE caused by virulent organisms such as *Staphylococcus aureus*. Rapid valvular destruction may precede the development of an audible regurgitant murmur in the early phase of illness, particularly when the hemodynamic consequence of regurgitation is masked by the patient's overall critical state [5,6]. Furthermore, acute severe valvular regurgitation characteristically produces atypical, soft, or abbreviated murmurs. In acute severe AR, rapid equalization of aortic and left ventricular diastolic pressures truncates the diastolic gradient, shortening and softening the early-diastolic murmur; in acute severe mitral regurgitation, rapid elevation of left atrial pressure diminishes the systolic pressure gradient and abbreviates the systolic murmur. In a tachycardic, tachypneic patient receiving non-invasive or invasive ventilation, these subtle findings are readily missed on initial auscultation. The respiratory predominance of symptoms naturally directed initial clinical assessment and investigation towards primary pulmonary pathology. The concurrent active EBV infection, with its attendant systemic constitutional features and bilateral pulmonary infiltrates, provided a seemingly plausible alternative explanation for an ARDS-like presentation [7,8]. Importantly, no outpatient cardiac assessment had been undertaken during the preceding weeks of constitutional illness, representing a potential missed opportunity for earlier cardiac evaluation. The rapid deterioration following extubation, however, provided a critical diagnostic window: the emergence of new auscultatory findings on serial examination prompted immediate echocardiography and established the diagnosis. This case reinforces that serial cardiovascular reassessment is as important as the initial examination in critically ill patients.

Several mechanisms could plausibly account for the initial respiratory presentation, and objective differentiation between them was not achievable at admission. The clinical picture could represent (a) acute severe valvular regurgitation from established IE producing cardiogenic pulmonary edema mimicking ARDS; (b) primary staphylococcal pneumonia with concomitant bacteremia seeding the valves, with IE-related valvular regurgitation emerging subsequently; or (c) a combination of both processes. The Berlin definition of ARDS does not exclude a cardiogenic contribution where an ARDS risk factor is present, and NT-proBNP, which in retrospect might have helped discriminate these possibilities, was not measured on admission. Admission bedside echocardiography was also not performed prior to the first intubation, representing a missed opportunity for earlier cardiac evaluation. Several features nonetheless argue against mechanism (b) alone: admission blood cultures were positive for *S. aureus* (rather than being a late finding); sputum cultures were negative; and by day 5, there were large mobile vegetations on both the aortic and mitral valves with severe bivalvular regurgitation, a trajectory too rapid for de novo vegetation formation and bivalvular destruction from a newly acquired pneumonia-related bacteremia. The outpatient PET-CT performed two weeks before admission was neither cardiac-gated nor targeted for endocarditis assessment, and 18F-FDG PET/CT has limited sensitivity for native-valve IE compared with its established role in prosthetic-valve IE [2].

Identification of a plausible portal of entry is an important component of the clinical workup in *S. aureus* IE, particularly in young patients without conventional cardiac risk factors or a history of intravenous drug use. In this case, the left cervical lymph node biopsy performed approximately two weeks prior to admission warrants consideration as a possible source of transient bacteremia, although a definitive causal relationship cannot be established retrospectively [2]. The patient denied any recent dental procedures, and admission examination revealed no cellulitis, cutaneous abscess, or other overt skin or soft tissue infection. Percutaneous and surgical procedures involving the skin and subcutaneous tissues are recognized as potential portals of entry for *S. aureus*, which colonizes the skin in a significant proportion of the general population [11]. The concurrent active EBV infection may have further facilitated IE following bacteremia, given its known immunomodulatory effects, including impaired T-cell function and altered innate immune responses [7,8]. The direct causal relationship between active EBV infection and susceptibility to IE requires further mechanistic investigation. HIV 1/2 serology was negative, and the patient had no clinical, laboratory, or historical features suggesting underlying immunocompromise. EBV-related lower respiratory tract involvement sufficient to account for an ARDS-like radiographic picture is uncommon in the immunocompetent host.

The simultaneous involvement of both the aortic and mitral valves is an uncommon but recognized complication of left-sided IE. The anatomical substrate for this contiguous spread is the aortomitral curtain, the fibrous continuity between the posterior aortic root and the anterior mitral valve leaflet. This provides a direct pathway for infection to extend from one valve to the other [2]. IE affecting the aorto-mitral continuity carries a particularly adverse prognosis, as it complicates surgical repair and may require more extensive reconstruction. Clinicians should anticipate dual-valve involvement when large vegetations are identified on either the aortic or mitral valve in left-sided IE, as this significantly influences surgical strategy and intraoperative planning [2,12].

Early echocardiographic assessment proved pivotal in this case, both in confirming the diagnosis and guiding surgical management. The 2023 ESC Guidelines for the management of IE designate TTE as a Class I, Level B investigation in all patients with suspected IE, with TOE recommended in addition when TTE is inconclusive or when complications are suspected [2]. The quantitative echocardiographic parameters employed, EROA and regurgitant fraction, are those endorsed by international guidelines for grading the severity of valvular regurgitation [9,12]. It is important to note that the modified Duke criteria classify 'vascular phenomena' as major arterial emboli, septic pulmonary infarcts, mycotic aneurysms, intracranial hemorrhage, conjunctival hemorrhages, and Janeway lesions [10]. Cardiogenic pulmonary edema and non-specific pulmonary infiltrates are not encompassed within this definition and should not be miscategorized as vascular phenomena. In the present case, definite IE was established by two major criteria alone, without the need for minor criteria.

The 2023 ESC Guidelines recommend urgent surgery (within 24-72 hours) as a Class I indication in patients with IE complicated by heart failure caused by severe valvular regurgitation [2]. The 2020 ACC/AHA Guidelines similarly support early surgical intervention in IE complicated by hemodynamically significant valvular dysfunction refractory to medical therapy [12]. Our patient fulfilled this indication, and a timely surgical referral was life-saving. Regarding prosthesis selection, mechanical valves are preferred for patients under 50-60 years of age owing to superior durability and freedom from structural valve deterioration, accepting the requirement for lifelong anticoagulation with warfarin. Current evidence supports this approach in young patients, and international guidelines endorse shared decision-making with consideration of patient preferences, lifestyle, and anticoagulation suitability [12]. The additional risk of endocarditis on mechanical prostheses, prosthetic valve endocarditis, should be discussed with patients as part of long-term follow-up [2].

IE, presenting primarily as ARF without overt cardiac signs is reported in a limited number of published cases. Davis et al. described a case of *S. aureus* IE masquerading as septic shock with ARDS, in which respiratory failure dominated the clinical picture and the cardiac etiology was identified only after significant clinical deterioration [5]. Sadi and Mansouri similarly reported IE presenting primarily as acute respiratory distress, with diagnosis established following failure to improve with standard ARDS management [4]. E.D. et al., in a case series addressing atypical IE presentations, highlighted the breadth of non-classical features, including isolated respiratory syndromes, and emphasized the need for heightened clinical suspicion and early echocardiography [3]. Tsolaki et al. further discussed the diagnostic complexity of distinguishing IE from non-IE in atypical presentations, reinforcing the importance of integrating clinical, microbiological, and echocardiographic data [8]. Collectively, these cases and the present report support early echocardiography as a core investigative tool in all patients with unexplained ARF and positive blood cultures.

## Conclusions

This case demonstrates that IE can present as ARF closely mimicking ARDS, particularly when caused by virulent organisms such as *Staphylococcus aureus*, producing acute severe valvular regurgitation. The absence of a cardiac murmur or peripheral stigmata of IE on initial examination does not exclude the diagnosis; serial cardiovascular reassessment and early bedside echocardiography are essential in all patients with unexplained ARF and bacteremia. Cardiogenic pulmonary edema from acute severe valvular regurgitation can closely mimic ARDS, and avoidance of injudicious fluid resuscitation is critical in septic patients with respiratory failure. Prior invasive procedures, such as a lymph node biopsy, should be considered as potential portals of entry for bacteremia in young patients with IE and no conventional cardiac risk factors. Simultaneous aortic and mitral valve involvement may result from contiguous spread via the aortomitral curtain and should be anticipated when planning surgical strategy.

In young patients with IE requiring valve replacement, bilateral mechanical prostheses offer superior long-term durability; patients must be counselled regarding lifelong anticoagulation and the risk of prosthetic valve endocarditis. Early multidisciplinary involvement and adherence to ESC and ACC/AHA guideline criteria for urgent surgical intervention in hemodynamically significant IE are life-saving. This case reinforces that early echocardiographic evaluation should be considered a mandatory component of the workup for any patient with unexplained respiratory failure, new bacteremia, or clinical features that do not fit a straightforward pulmonary diagnosis.
